# Partial in-vitro dispersal of *S. mutans* UA159 biofilms by silver-(I)cyanoximate compounds.

**DOI:** 10.17912/micropub.biology.001262

**Published:** 2024-08-12

**Authors:** Brendaliz Santiago Narvaez, Sarah Hameer, Jamie L. Perry, Tiffany Rojas, Laurel G. Habgood

**Affiliations:** 1 Biology, Rollins College, Winter Park, Florida, United States; 2 Chemistry, Rollins College, Winter Park, Florida, United States

## Abstract

Silver(I) cyanoximate compounds have antibacterial activity against the oral pathogen *Streptococcus mutans,* a resident of oral plaque biofilm. As oral biofilm strategies focus on the inhibition of attachment or physical removal of the existing microbes, we were interested in exploring the ability of six different silver(I) cyanoximate compounds to target and disperse a pre-existing biofilm. Here we report that these compounds were only able to partially disperse *S. mutans* biofilms as the compounds were more effective at inhibiting biofilm formation. None of the six compounds were able to outperform silver nitrate, a commonly used antibacterial in dentistry.

**Figure 1. Partial in-vitro dispersal of S. mutans UA159 biofilms by silver (I) cyanoximate compounds.**
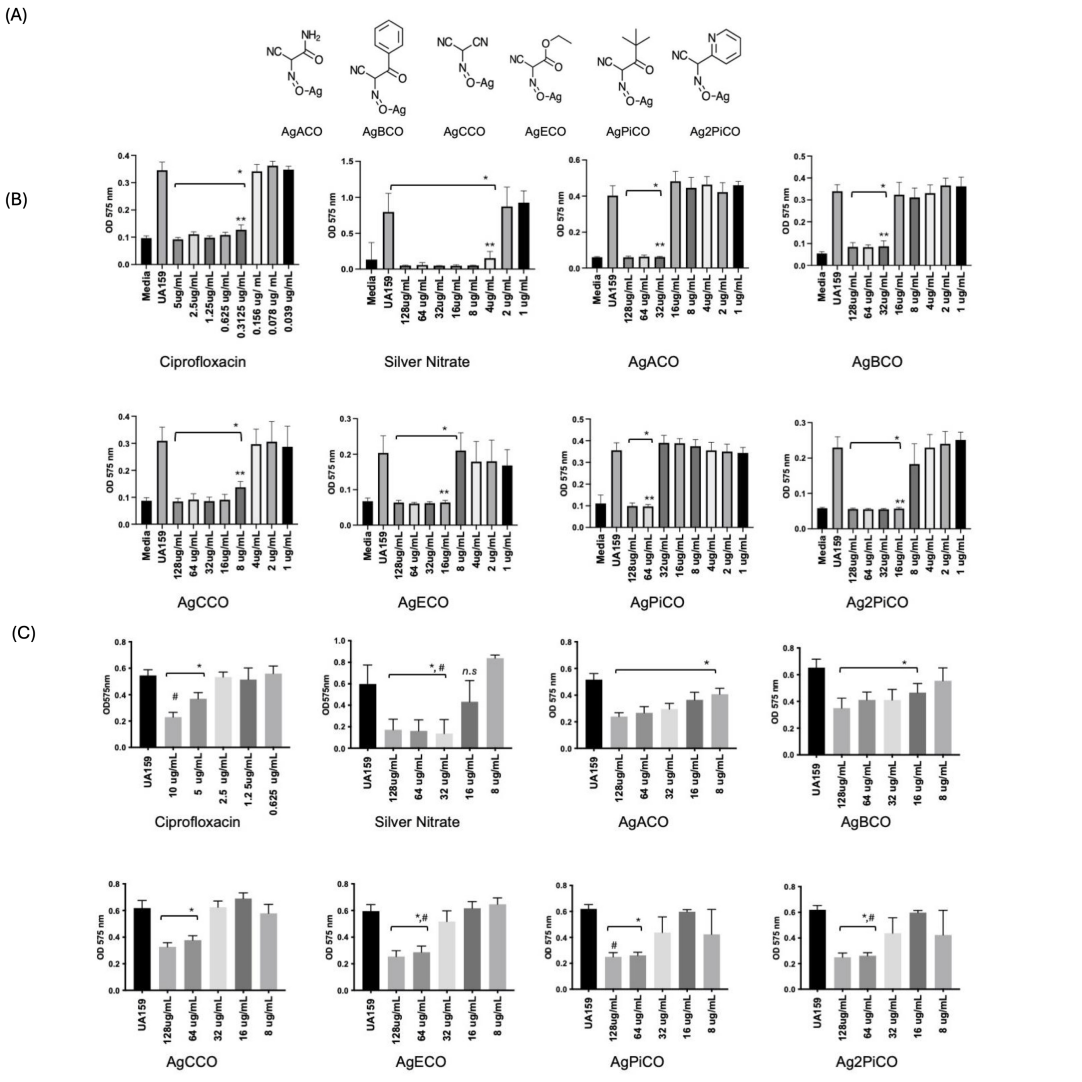
(A)Chemical structures of the six tested silver(I) cyanoximate compounds. silver(I)a-oximido(acetamide)acetonitrile (AgACO), silver(I)a-oximido-(2-benzoyl) acetonitrile (AgBCO), silver(I)nitrosodicyanomethanide (AgCCO), silver(I)a-oximido-(ethylacetoxy)acetonitrile Ag(ECO), silver(I)a-oximido-(2 pivaloyl)acetonitrile (AgPiCO) and silver(I)a-oximido-(2-pyridyl)acetonitrile (Ag2PiCO). (B) Minimum Biofilm Inhibitory Concentrations (MBIC) for biofilm cultures of S.mutans UA159 grown in in TYS 1% (w/v) sucrose in presence of silver(I) cyanoximates. Significant difference for MBIC was determined using student's t-test with * indicating a statistically significant value (p-value < 0.0001) from WT value. ** indicate MBIC values. MBIC determined by OD cut off (ODc) value three standard deviations above mean OD 575 of negative control; ODc= AVG OD Negative control + (3X SD Negative control), n=8. (C) Biofilm dispersal of S. mutans UA159. Twenty-four-hour biofilms grown in TYS 1% (w/v) sucrose followed by treatment with compounds. Plates were incubated for an additional 24 hours where supernatant was removed, followed by staining with 0.1% (w/v) crystal violet. Crystal violet was extracted with 30%(v/v) acetic acid. Absorbance was measured with a microplate reader at 575 nm. Statistical significance in biofilm disruption assays was determined by Student’s t-test; * *p < 0.001; n=8. #, indicates the concentration of compound where biofilm reduction was > 50% as compared to untreated (WT) biofilm. Error bars represent standard deviation.

## Description

The oral cavity contains hundreds of microbial species that collectively make up the oral microbiome (Baker & Edlund, 2019) . Although dental plaque consists of a multi-species biofilm, Streptococcus mutans, a Gram-positive organism, continues to be a key contributing species in the development of human dental caries (Scharnow et al., 2019). Biofilm formation, aciduricity and acidogenicity are three of S. mutans’ key virulence properties that lead to the alteration of the oral microenvironment, thus making it more favorable for pathogenic species (Lemos et al., 2019; Sauer et al., 2022) . Biofilms are inherently resistant to antimicrobial agents, therefore many of the existing antibiofilm strategies focus on inhibition of attachment and or physical removal of the biomass (Barraud et al., 2015; Z. Gao et al., 2024; Scharnow et al., 2019; ten Cate, 2006) . Inhibiting the formation of a biofilm is a critical first step in the prevention of biofilm associated infections (Scharnow et al., 2019). The use of silver in surface modification (“coating”) has been the focus of much research to reduce microbial colonization and biofilm formation (Möhler et al., 2018). Silver compounds prevent attachment, a pivotal step of biofilm formation (Eckhardt et al., 2013; Kalishwaralal et al., 2010) . In dentistry, silver nitrate is commonly used as an antimicrobial against oral pathogens(S. S. Gao et al., 2018; Spacciapoli et al., 2001). Silver diamide fluoride and silver nanoparticles have been strongly suggested as effective caries preventative interventions (Martínez-Robles et al., 2016; Rosenblatt et al., 2009) . Among the newer silver-based antimicrobials studied are silver(I) cyanoximates, which consist of silver salts and oxime-based ampolydentate ligands (Gerasimchuk et al., 2010; Lotlikar et al., 2019) . Biofilms of P. aeruginosa PAO1, S. aureus NRS70, and S. mutans UA159 are inhibited when these compounds are incorporated into composite materials (Riddles et al., 2014) . As dental plaque is typically already present over the surface of the tooth, we wanted to test the ability of these compounds to target a pre-existing S. mutans UA159 biofilms. To further examine the antibiofilm properties of six silver (I) cyanoximates (Fig1A), we determined minimum biofilm inhibitory concentrations (MBIC) and measured biofilm dispersal by these compounds in comparison to silver nitrate, a commonly used antimicrobial in dentistry. As inhibition of biofilm formation was already established for the compounds incorporated in composite materials (Riddles et al., 2014) ; we were interested in determining minimum biofilm inhibitory concentrations (MBIC) for all six compounds when these were added to biofilm cultivation medium (TY1%(w/v) sucrose). Sucrose is an important carbohydrate as S. mutans utilizes this carbon source for the formation of EPS (glucans) in plaque (Forssten et al., 2010; Jijakli & Jensen, 2019) . MBIC assays confirmed that the compounds were able to inhibit S. mutans biofilms in-vitro (Fig.1B) (Riddles et al., 2014) . The concentrations needed to inhibit biofilms were typically higher than those reported for the inhibition of planktonic cultures (Gerasimchuk et al., 2010) . The requirement of higher concentrations of a drug to target a biofilm is not surprising due to the resistant nature of biofilms(Grande et al., 2020; Høiby et al., 2010) . This behavior has been reported for silver (I) cyanoximates used against Gram-positive organisms including S. mutans UA159 (Riddles et al., 2014) . Compounds that were the most effective at preventing biofilm formation were AgCCO, AgECO and Ag2PiCO (Fig.1B). Previous studies showed that AgPiCO and its derivatives had the most stable inhibition against biofilms (Lotlikar et al., 2019; Riddles et al., 2014) . The compounds, however, did not outperform established antimicrobials, as inhibition of biofilm formation by ciprofloxacin and silver nitrate occurred at exceedingly lower concentrations (> 0.3125 ug/mL and 4 ug/mL respectively). Our results demonstrate that although silver(I)cyanoximate compounds were able to inhibit S. mutans biofilms, as previously reported, silver nitrate is more effective. Destruction of an already established biofilm is one of the more difficult aspects of biofilm-based infections. Biofilms are challenging for antibiotics to penetrate due to their density and structure, therefore a favorable characteristic of an antibacterial lies in its ability to destroy a preformed biofilm (Kaplan, 2010) . The eradication of established biofilms on enamel have been tested with chlorhexidine mouth rinses and support the consideration of biofilm disrupting agents in oral biology(Martínez-Hernández et al., 2020; Seguya et al., 2022). We next measured the biofilm dispersal of the compounds against S. mutans. Biofilm dispersal assays consisted of growing S. mutans UA159 biofilms for 24 hours, followed by treatment with the compounds. Our biofilm dispersal assays showed that the compounds could only partially disperse the 24-hour biofilms, and that their ability to do so was limited in comparison to silver nitrate or ciprofloxacin (Fig 1C). The most significant reduction of biofilm mass observed (>75% in comparison to WT) occurred in presence of silver nitrate (32ug/mL). For the silver cyanoximates, the highest dispersal (>50%) occurred at ranges equal to or above the established MBIC (>64ug/ml) (Fig 1C). Our data indicates that dispersal requires higher concentrations for the compounds to have a significant effect. The six tested silver(I) cyanoximate compounds contain different ligands. These structural differences could contribute to the exhibited differences in their ability to interfere with attachment and biofilm formation in S. mutans UA159. Compounds exhibited different behaviors between biofilm inhibition and biofilm dispersal characteristics. For example, AgACO and AgBCO and AgPiCO were able to disperse biofilms at concentrations lower than those required for them inhibit biofilm formation. Compounds with hydrophobic aromatic ring structures; like those present in AgBCO and Ag2PiCO, have been shown to interfere with quorum sensing, a pivotal process in S. mutans biofilm formation and maturation(Krzyściak et al., 2014). Ag2PiCO's inhibition of biofilm formation and AgBCO’s ability to destroy preformed biofilms at lower concentrations suggest that this ligand structure may be inhibiting interactions crucial for biofilm formation. Our work confirms that silver(I) cyanoximate compounds have antimicrobial properties against the oral pathogen S. mutans UA159, however, their effectiveness was limited in comparison to silver nitrate. Additionally, the amount required to inhibit or disperse in vitro biofilms with the silver(I) cyanoximates was high, which points at the necessity to conduct toxicity studies if these compounds are to be used in solution or via the incorporation into composite material (Lansdown, 2010; Zhou et al., 2023) .

## Methods

Methods: Bacterial strains and culture conditions S. mutans UA159 (Ajdić et al., 2002) was struck out onto Brain Heart Infusion (BHI) (Sigma-Aldrich, St. Louis, MO) agar to obtain single, isolated colonies. Plates were incubated for 24-48 hours at 37°C in a 5% (v/v) CO2/ 95% air atmosphere. An isolated colony was then suspended into BHI broth and incubated for 24 hours to generate overnight cultures. For biofilm assays, S. mutans UA159 was grown in Tryptone Yeast (TY) Medium (Sigma-Aldrich, St. Louis, MO) supplemented with 1% (w/v) sucrose (TYS). Compounds and reagent stocks The six tested silver(I)cyanoximate (silver cyanoximates) compounds used in this study were silver(I)a-oximido(acetamide)acetonitrile (AgACO), silver(I)a-oximido-(2-benzoyl)acetonitrile (AgBCO),silver(I)nitrosodicyanomethanide (AgCCO), silver(I)a-oximido-(ethylacetoxy)acetonitrile Ag(ECO), silver(I)a-oximido-(2 pivaloyl)acetonitrile (AgPiCO) and silver(I)a-oximido-(2-pyridyl)acetonitrile (Ag2PiCO)(Young et al., 2021). These compounds were synthesized following as previously described (Gerasimchuk et al., 2010) . All compounds were resuspended in dimethyl sulfoxide (DMSO) (Sigma-Aldrich, St. Louis, MO) to create 1 mg/mL stock solutions. Two-fold serial dilutions were performed (ranging from 128 µg/mL to 0.25 µg/mL) for each compound to be used for MBICs, and biofilm dispersal assays. Silver nitrate (1mg/mL) (Sigma-Aldrich, St. Louis, MO) and Ciprofloxacin (200 ug/ml) (Sigma-Aldrich, St. Louis, MO) stock solutions were used for control experiments. Minimum Biofilm Inhibitory Concentration (MBIC) Assay Minimum Biofilm Inhibitory Assays were performed as previously described (Saputo, S, R C Faustoferri, 2018) . MBIC assays were performed similarly to MIC assays in 96-well plates (Wiegand et al., 2008) . Overnight cultures were diluted 1:100 in tryptone yeast plus 1% (w/v) sucrose (TYS). After inoculation, the 96-well plates were incubated overnight at 37°C in a 5% (v/v) CO2/ 95% air atmosphere. The next day, the supernatant was removed, the wells washed with sterile distilled water, and allowed to dry overnight. Biofilms were then stained with 0.1% (w/v) crystal violet (Sigma-Aldrich, St. Louis, MO), washed with distilled water, and dried. To solubilize the crystal violet, 30% (v/v) acetic acid was added to all wells. Solubilized crystal violet was transferred to new microtiter plates and the absorbance measured with a microplate reader at 575 nm (BioTek Synergy HTX Multi-Mode Reader, Winooski, VT), using acetic acid as the blank. Minimum biofilm inhibition values were determined by identifying culture conditions three standard deviations above the mean OD of negative control (Stepanović et al., 2007). Biofilm Dispersal Assay Biofilm disruption assays were performed as described by Chen et al. with modifications (Chen et al., 2016) . S. mutans UA159 overnight cultures were normalized to 0.3 in TYS medium and used to inoculate 96-well plates with fresh TYS 1 % (w/v) sucrose medium. Plates were incubated at 37°C in a 5% (v/v) CO2/ 95% air atmosphere to allow for biofilm growth over a 24-hour period. The next day, the supernatant was aseptically removed, and plates inoculated with fresh TYS media containing increasing concentrations of each tested compound ranging from 8µg/mL to 128 µg/mL. Plates were incubated for an additional 24 hours. After incubation, the supernatant was removed, and the wells washed three times with sterile distilled water and allowed to dry for a few hours. The remaining biofilms were stained with 0.1% (w/v) crystal violet, washed with distilled water, and left to dry for another few hours. Crystal violet was solubilized with 30% (v/v) acetic acid. The solubilized crystal violet was transferred to new microtiter plates and absorbance was measured with a microplate reader at 575 nm (BioTek Synergy HTX Multi-Mode Reader), using acetic acid as the blank. Statistical Analysis: All analysis was done using GraphPad Prism version 10.1.0 for iOS, GraphPad Software, Boston, Massachusetts USA (Motulsky, 2022) .
